# Septic arthritis by *Sphingobacterium multivorum* in immunocompromised pediatric patient

**DOI:** 10.1016/j.rppede.2016.03.014

**Published:** 2016

**Authors:** Maiana Darwich Mendes, Rafael Ruiz Cavallo, Cecilia Helena Vieira Franco Godoy Carvalhães, Maria Aparecida Gadiani Ferrarini

**Affiliations:** a Escola Paulista de Medicina da Universidade Federal de São Paulo (Unifesp), São Paulo, SP, Brazil

**Keywords:** Arthritis, Sphingobacterium, Bacteria

## Abstract

**Objective:**

To report a case septic arthritis with a rare pathogen in a immunosuppressed child.

**Case description:**

Male patient, 6 years old, had liver transplant five and half years ago due to biliary atresia. Patient was using tacrolimus 1mg q.12h. This patient started to have pain in left foot and ankle and had one episode of fever 3 days before hospital admission. Physical examination showed weight 17kg, height 109cm, temperature 36.4°C, with pain, swelling and heat in the left ankle, without other clinical signs. Initial tests: hemoglobin 11.7g/dL hematocrit 36.4%, leukocyte count 17,600µL^-1^ (7% banded neutrophils, 70% segmented neutrophils, 2% eosinophils, basophils 1%, 13% lymphocytes, 7% monocytes) C-reactive protein 170.88mg/L. Joint ultrasound showed moderate effusion in the site. Patient was submitted to surgical procedure and *Sphingobacterium multivorum* was isolated from the effusion. The germ was susceptible to broad spectrum cephalosporins (ceftriaxone and cefepime) and fluoroquinolones (ciprofloxacin and levofloxacin), and it was resistant to carbapenemic antibiotics and aminoglycosides. He was treated intravenously with oxacillin for 15 days and ceftriaxone for 13 days, and orally with ciprofloxacin for 15 days, with good outcome.

**Comments:**

The *S. multivorum* is a gram negative bacillus that belongs to Flavobacteriaceae family and it is considered non-pathogenic. It has rarely been described as a cause of infections in humans, especially in hospital environment and in immunosuppressed patients. This case report is relevant for its unusual etiology and for the site affected, which may be the first case of septic arthritis described.

## Resumo

**Objetivo:**

Relatar um caso de artrite séptica de etiologia rara em uma criança imunossuprimida.

**Descrição do caso:**

Paciente masculino, seis anos, transplantado hepático havia cinco anos e meio devido à atresia de vias biliares, em uso de tacrolimus 1 mg de 12/12 horas, iniciou dor em pé e tornozelo esquerdo e um episódio de febre três dias antes da internação. Ao exame físico, peso 17kg, estatura 109cm, temperatura de 36,4°C, com dor, edema e calor no tornozelo esquerdo e sem outras alterações. Exames da entrada: hemoglobina 11,7g/dL, hematócrito 36,4%, leucócitos, 17.600/uL (7% bastões, 70% segmentados, 2% eosinófilos, 1% basófilo, 13% linfócitos, 7% monócitos), proteína C reativa 170,88mg/L. Ultrassonografia articular evidenciou moderado derrame no recesso tíbio talar anterior esquerdo. Feita limpeza cirúrgica com o isolamento do *S. multivorum* na cultura do líquido articular, suscetível a um amplo espectro de cefalosporinas (cefepime e ceftriaxone) e fluoroquinolonas (ciprofloxacino e levofloxacino), esistente a carbapenêmicos e aminoglicosídeos. Tratado com oxacilina por 15 dias e ceftriaxone 13 dias intravenoso e ciprofloxacina via oral por mais 15 dias com boa evolução.

**Comentários:**

O *Sphingobacterium multivorum* é um bacilo gram negativo, pertencente à família *Flavobacteriaceae*, considerado não patogênico, tem sido raramente descrito como etiologia de infecções em seres humanos principalmente em ambientes hospitalares e em imunossuprimidos. O relato deste caso é relevante por sua etiologia incomum e pelo sítio acometido, pode ser este o primeiro caso de artrite séptica descrito.

## Introduction

Septic arthritis is caused by the presence of a pathogenic microorganism in the joint space and represents a diagnostic and therapeutic challenge. It affects mainly children and *Staphylococcus aureus* is the most common etiological agent. The implementation of early and appropriate treatment is essential for a favorable evolution without sequelae.^1^

Unusual etiologies of septic arthritis have been reported, also in immunocompetent children, as in the case described in India, from which Achromobacter xylosoxidans was isolated,^2^ but immunosuppression is a determining factor regarding the presence of other etiological agents rather than *S. aureus* and unfavorable evolution.

Immunocompromised patients are more likely to develop infections with unusual etiologies, such as *Mycoplasma hominis*, which has been associated with septic arthritis in the immunosuppressed pediatric population.^3^ In these patients, the diagnosis is often delayed, which can determine the evolution to erosive arthritis, joint space destruction and sepsis.^4^

*Sphingobacterium multivorum* is a gram-negative, saprophytic bacillus of the Flavobacteriaceae family, naturally found in soil, plants and water,^5^ first described in 1981.^6^ It was considered nonpathogenic for a long time, but for some years now it has been described as a cause of infectious processes in human beings.^7^

The objective of this study is to report the case of an immunosuppressed pediatric patient who developed septic arthritis by *S. multivorum*.

## Case description

A six year-old male patient was admitted to the Pediatric Emergency Room with a history of pain in the left foot and ankle, together with difficulty in ambulation for five days, with reports of an isolated fever peak (39°C) three days before admission.

At admission, his weight was 17kg, height 109cm, body mass index of 14.3, heart rate of 120bpm, blood pressure of 95×62mmHg, temperature of 36.4°C, with swelling and warmth in the left ankle and mild pain at mobilization. The remaining physical examination was uneventful.

The patient was born at 39 weeks, by cesarean section. The mother reported an uneventful prenatal period. On the second day of life the patient had jaundice and was submitted to phototherapy for eight days. It progressed without improvement and he was referred for outpatient treatment. At three months of age, he was diagnosed with biliary atresia and at six months he was submitted to liver transplantation. He has received immunosuppressive medication since then (currently receiving tacrolimus, 1mg q.12hs). Due to the presence of some phenotypic deviations and single kidney on the right, he is also followed by the genetics discipline of the same institution, but still without a diagnosis. He has a normal karyotype. He is being followed at the Child Development Outpatient Clinic of the same service and all his vaccines are up-to-date for his age.

Laboratory tests at admission: hemoglobin 11.7g/dL, hematocrit 36.4%, white blood cell count of 17,600µL^-1^ (7% band cells, 70% segmented, 2% eosinophils, 1% basophils, 13% lymphocytes, 7% monocytes) and C-reactive protein, 170.88mg/L. The ultrasonography showed moderate joint effusion in the left anterior tibiotalar recess.

The patient was admitted and intravenous therapy with oxacillin 200mg/kg/day was started. Two days after admission, the patient underwent surgical cleaning of the joint together with drainage of secretions, which were sent to culture without other joint fluid analysis. After two days, the partial result showed growth of gram-negative bacilli and ceftriaxone (100mg/kg/day) was associated to oxacillin.

The joint fluid culture showed bacterial growth on blood agar, in which they appeared as non-hemolytic colonies of 1mm in diameter, convex, smooth, opaque after 48h-incubation at 37°C. Bacterial identification was carried out with the Phoenix^®^ automated system, which identified *Sphingobacterium multivorum* (90% certainty), with negative results for the L-glutamic acid test. Additional tests, such as the positive response to the oxidase and urease tests and morphological characteristics and sensitivity profile confirmed the identification of the bacterium.

Antimicrobial susceptibility was tested using the Phoenix^®^ automated system and interpreted with the CLSI document (M100S25).^8^ The isolated bacteria were susceptible to a broad spectrum of cephalosporins (ceftriaxone and cefepime) and fluoroquinolones (ciprofloxacin and levofloxacin). Nevertheless, it showed a resistant phenotype to carbapenems (imipenem and meropenem; minimum inhibitory concentration (MIC) >8mg/L) and aminoglycosides (Table 1).

**Table 1:** Sensitivity profile of the isolated strain in the case report.

Result	Antibiotic and MIC
Sensitive to	Cefepime (MIC 4), ceftriaxone (MIC 4), levofloxacin (MIC≤1), sulfamethoxazole-trimethoprim (MIC ≤0.5), ciprofloxacin (MIC 1)
Resistant to	Tobramycin (MIC>8), amikacin (MIC>32), gentamicin (MIC>8), meropenem (MIC>8), piperacillin-tazobactam (MIC>64), imipenem (MIC>8), aztreonam (MIC>16), cefotaxime (intermediate resistance MIC 32)

Central Laboratory of the hospital where the study was carriedout. MIC (minimum inhibitory concentration): in mg/mL.

Treatment was maintained, as the patient was in good overall status, with no fever, no signs of inflammation in the joint and improvement in inflammatory tests and white blood cell count. On the 13th day of oxacillin and 11th day of ceftriaxone use, he showed increased liver transaminase levels (aspartate transaminase (AST) 115U/L and alanine transaminase (ALT) 114U/L, respectively) and pulse therapy was started (10mg/kg methylprednisolone) for two days with decrease in liver enzymes levels (AST: 43U/L and ALT: 79U/L).

He was discharged after 15 days of amoxicillin and 13 days of ceftriaxone, with maintenance of oral treatment with ciprofloxacin for 15 days, according to the antibiogram. At discharge, he showed no signs of inflammation in the affected joint and CRP was 2.8mg/L.

He returned to outpatient care 12 days after discharge, still receiving ciprofloxacin 250mg q.12hs. At the time, he was asymptomatic and had no complaints. The antibiotic was maintained for three more days. At the following consultation, 15 days later, he was clinically well and a new measurement of CRP was <0.6mg/L, one week after the end of treatment. At the monthly follow-up, the patient remained asymptomatic, with no signs of inflammation in the affected joint. He remains in outpatient treatment, with a follow-up schedule for at least one year.

## Discussion

This is a patient with septic arthritis caused by a bacterium that rarely causes infections in humans and there has been no description of pyoarthritis with this etiology to date. The *Sphingobacterium multivorum* initially was not considered pathogenic, but in recent years it has been related to infections, especially in-hospital ones and in immunosuppressed patients.^7^

There has been a description of this agent not only in hospitals but also as a contaminant of public transport objects in the city of São Paulo, with resistance to cephalexin and cefazolin.^9^ The presence of this bacterium in the environment has already allowed its inoculation in a prostate biopsy procedure.^10^ That demonstrates that *S. multivorum* is present in the environment and may be an infectious agent, mainly in immunosuppressed individuals.

In 1999, this bacterium was primarily related to opportunistic infection in patients with the human immunodeficiency virus (HIV)^7^ and was later described as the triggering factor of sepsis and death in a patient with this virus.^11^ *S. multivorum* has also been associated to sepsis under other circumstances, such as the duration of chemotherapy,^12^ hemodialysis^13^ and in an elderly patient with chronic obstructive pulmonary disease, hypertension and diabetes^14^ ; and in the airway colonization of patients with cystic fibrosis,^15^^,^ 16 but case reports of arthritis caused by this bacterium were not found in the literature. The patient's age range is also noteworthy, as most infection reports by this microorganism describes the involvement of adults and elderly patients with comorbidities.^8^^,^ 11 ^-^ 16 In 2006, the first case of sepsis caused by *S. multivorum* was described in Turkey, in a previously healthy pediatric patient, who was treated with ampicillin and cefotaxime and progressed with full recovery without complications.^17^ A summary of the 17 cases reported in the literature is shown in Table 2.

**Table 2:** Summary of reports of infections caused by *S. multivorum*.

Author and reference	Patient's age/site of infection/comorbidity	Treatment	Evolution
Areekul^11^	47 years/sepsis/HIV and diabetes	Gentamicin and ampicillin. Subsequently, ceftriaxone and sulfamethoxazole-trimethoprim	Death
Freney^12^	57 years/sepsis/non-Hodgkin's lymphoma	Pefloxacin and sulfamethoxazole-trimethoprim	Full recovery
Lambiase^15^	3 positive samples in 332 patients with cystic fibrosis and chronic pulmonary infection	No data	No data
Aydoğan^17^	2 months/sepsis/healthy	Ampicillin and cefotaxime full recovery	Full recovery
Ramírez^18^	74 years/pneumonia/chronic obstructive pulmonary disease	Ceftazidime, cefuroxime.	Full recovery
Grimaldi^5^	64 years/septic shock/obesity and coronary disease	Amoxicillin and clavulanate	Full recovery
Nielsen^10^	Study with 3 patients after prostate biopsy. Patient 1:79 years/cystitis Patient 2: 59 years/cystitis Patient 3: 69 years/cystitis	1- Piperacilina-tazobactam and ciprofloxacin 2- No antibiotics 3- Sulfamethoxazole-trimethoprim	Full recovery
Potvliege^13^	43 years/sepsis/on hemodialysis	Ampicillin	Full recovery
Reina^16^	1 year and 8 months/cystic fibrosis	Ceftazidime and amikacin	Full recovery
Barahona^14^	67 years/sepsis/obesity, arterial hypertension and chronic obstructive pulmonary disease	Cefepime and vancomycin, ciprofloxacina	Full recovery

Another point to be emphasized is the importance of adequate antimicrobial therapy to be implemented in such cases. The susceptibility profile of the strain described in this case is in accordance with a study by Lambiase et al., with a carbapenem and aminoglycoside resistance profile^15^ ; however there is an account of respiratory infection by *S. multivorum* in which a strain sensitive to Imipenem was demonstrated.^18^ The present study showed sensitivity to third-generation cephalosporins. However, other reports showed resistance to these antibiotics,^14^^,^ 15 including a study that showed the capacity of the bacterium to cause hydrolysis of third-generation cephalosporins and carbapenems.^19^

In this case, both ciprofloxacin and sulfamethoxazole-trimethoprim could be used. However, the choice of ciprofloxacin for oral therapy after hospital discharge was due to the fact that the etiological agent had not yet been described as the cause of septic arthritis in our country, due to its morphological similarity to Pseudomonas,^20^ for which this antibiotic would be a good choice, as well as the patient's immunosuppression. Studies have shown that ciprofloxacin may be used in pediatric patients according to the analysis of risks and benefits in each situation.^21^^-^ 23 One of the indications by the American Academy of Pediatrics and the World Health Organization would be its use in bacterial infection by gram negative microorganisms in immunosuppressed children.^21^^,^ 24

It is noteworthy the relevance of the culture material, as it indicated the broader spectrum of the antimicrobial therapy, which allowed a favorable evolution of the patient's condition. Early diagnosis of arthritis also contributed to the absence of complications and sequelae.

Infections by *S. multivorum* have been studied more frequently in recent years, but studies in the literature involving this pathogen are still scarce. No articles were found on septic arthritis related to this bacterium, which makes it essential to report on the case of the studied patient.
